# First Description of Serological Evidence for SARS-CoV-2 in Lactating Cows

**DOI:** 10.3390/ani12111459

**Published:** 2022-06-04

**Authors:** Filomena Fiorito, Valentina Iovane, Ugo Pagnini, Claudia Cerracchio, Sergio Brandi, Martina Levante, Luisa Marati, Gianmarco Ferrara, Virginio Tammaro, Esterina De Carlo, Giuseppe Iovane, Giovanna Fusco

**Affiliations:** 1 Department of Veterinary Medicine and Animal Production, University of Naples Federico II, 80137 Naples, Italy; filomena.fiorito@unina.it (F.F.); ugo.pagnini@unina.it (U.P.); claudia.cerracchio@unina.it (C.C.); gianmarco.ferrara@unina.it (G.F.); 2 Department of Agricultural Sciences, University of Naples Federico II, Portici, 80055 Naples, Italy; valentina.iovane@unina.it; 3 Istituto Zooprofilattico Sperimentale del Mezzogiorno, Portici, 80055 Naples, Italy; sergio.brandi@izsmportici.it (S.B.); martina.levante@izsmportici.it (M.L.); luisa.marati@izsmportici.it (L.M.); giovanna.fusco@izsmportici.it (G.F.); 4 Azienda Sanitaria Locale Avellino (Ariano Irpino), 83031 Avellino, Italy; lupovick1966@gmail.com

**Keywords:** SARS-CoV-2, cattle, serology, COVID-19, farmworkers, reverse zoonosis

## Abstract

**Simple Summary:**

Severe Acute Respiratory Syndrome Coronavirus-2 (SARS-CoV-2) is the agent of the disease that has caused a global pandemic, known as coronavirus disease 2019 (COVID-19). Coronaviruses (CoVs) may emerge from wildlife hosts and infect humans and animals. Up to now, natural infection with SARS-CoV-2 has been reported in several animals, but it has not been found in farm animals, such as buffaloes, goats, sheep, horses, rabbits, hens, pigs, or cows, despite contact with their SARS-CoV-2-positive human breeders. Furthermore, a low susceptibility to SARS-CoV-2 has been detected in experimentally infected cattle with SARS-CoV-2. The unknown zoonotic potential of this virus is a cause of concern for pet owners and farmers. The limited data on cattle suggest that cattle show low susceptibility to SARS-CoV-2 and probably do not function as reservoirs. However, in areas with large cattle populations and a high prevalence of SARS-CoV-2 infection in humans, close contact between livestock and farmworkers may cause reverse zoonotic infections in cattle, as has already been described for highly sensitive animal species, such as minks, cats, and dogs. Thus, studying the zoonotic characteristics of SARS-CoV-2 could help in the development of a strategy for virus detection and the control of viral dissemination.

**Abstract:**

Following the COVID-19 epidemic outbreak in Ariano Irpino, Campania region (Italy), we tested lactating cows for the presence of SARS-CoV-2 on a cattle farm at which, prior to the investigation, 13 of the 20 farmworkers showed COVID-19-like symptoms, and one of them died. Twenty-four lactating cows were sampled to detect SARS-CoV-2. All nasal and rectal swabs and milk samples were negative for SARS-CoV-2 RNA. Of the 24 collected serum samples, 11 showed antibodies against SARS-CoV-2 nucleocapsid protein, 14 showed antibodies against SARS-CoV-2 spike protein, and 13 developed neutralising antibodies for SARS-COV-2; all samples were negative for Bovine Coronavirus (BCoV), another betacoronavirus. To our knowledge, this is the first report of natural serological evidence of SARS-CoV-2 infection in lactating cows. We hypothesise that this may be a case of reverse zoonosis. However, the role of cattle in SARS-CoV-2 infection and transmission seems to be negligible.

## 1. Introduction

The world is currently fighting a microorganism, SARS-CoV-2, the causative virus of the disease that has caused a global pandemic, known as COVID-19. SARS-CoV-2, a member of the family Coronaviridae, is a single-stranded enveloped RNA virus. Coronaviruses (CoVs), such as SARS-CoV-2, can emerge from wildlife hosts and infect humans and domestic and/or livestock animals [1,2,3]. Thus, they can cause epidemic or pandemic outbreaks, with low, medium, or high morbidity and mortality. Sequence analyses suggest that SARS-CoV-2 could have originated from bat CoVs, highlighting the interspecies transmission of CoVs [3,4,5,6], although the intermediate host is currently unknown.

Studying the zoonotic aspects of SARS-CoV-2 might aid in the development of a strategy for virus detection and the control of viral dissemination. To date, natural infection with SARS-CoV-2 has been reported in cats, dogs, minks, otters, pet ferrets, lions, tigers, pumas, snow leopards, gorillas, white-tailed deer, fishing cats, Binturong, South American coati, spotted hyenas, Eurasian lynx, Canada lynx, hippopotami, and hamsters **COVID-19-OIE-World Organisation for Animal Health**; [7,8,9,10,11,12,13].

However, it has not been detected in farm animals, such as buffaloes, goats, sheep, horses, rabbits, hens, pigs, or cows, despite contact with their SARS-CoV-2-positive human breeders for at least 2 weeks [14]. To date, a low susceptibility to SARS-CoV-2 has been detected in experimentally infected cattle with SARS-CoV-2. Indeed, the authors detected viral RNA in only two of the six inoculated animals, on day 3 after infection [15]. Furthermore, no active viral replication was detected in colostrum-deprived calves that were experimentally infected with SARS-CoV-2 [16]. The unknown zoonotic potential of this virus is a cause of concern for pet owners and farmers.

In April 2021, we investigated, for the presence of SARS-CoV-2 and BCoV, lactating cows on a farm at which 13/20 farmworkers in April 2020 had COVID-19-associated disease, and one of them had died. The farm was located in Ariano Irpino, the first city in the Campania region (Southern Italy) to be locked down and declared a red zone in March 2020.

This study is the first to describe the detection of SARS-CoV-2 antibodies in cattle. We hypothesise that this may represent a case of inverse zoonosis, i.e., human-to-animal transmission, which may lead to new reservoirs for the virus as well as the development of new viral variants that are potentially dangerous to humans and/or animals.

## 2. Materials and Methods

### 2.1. Samples

The sampling was carried out by the Local Official Veterinary Service during the Official Eradication Control Plane as specified in the Regulation (Eu) 2016/429 of the European Parliament and of the Council of 9 March 2016 on transmissible animal diseases and amending and repealing certain acts in the area of animal health (‘Animal Health Law’). Permission was obtained from the owners of the farm animals for collection of their nasal and rectal swab specimens. All methods were performed in accordance with the relevant guidelines and regulations.

The farm included 150 animals, of which 24 lactating cows were sampled (Table 1). The herd consisted of heifers, beef cows, lactating cows, and one bull, which died before sampling. To analyse also milk samples, we sampled only lactating cows. The herd had never been vaccinated against BCoV.

All samples were collected by local veterinary authorities. Nasal and rectal swab specimens were collected, frozen, and stored at −80 °C until processing. Blood was drawn from the jugular vein using sterile evacuated tubes without EDTA anticoagulant. Upon arrival at the laboratory, the samples were centrifuged, and aliquots of the serum and milk were frozen at −80 °C until testing.

### 2.2. Nucleic Acid Extraction

Nucleic acid extraction was performed in biosafety level 3 (BSL-3) laboratories. Aliquots (400 µL) of the milk samples collected from each cow were subjected to extraction and purification using the QIAsymphony DSP Virus/Pathogen Midi Kit (Qiagen, Hilden, Germany) and processed using the QIAsymphony automated system (Qiagen, Hilden, Germany) according to the manufacturer’s instructions, eluted in 60 µL, and stored at –80 °C until use.

Nucleic acid was extracted from nasal swabs as follows: 200 µL aliquots of Universal Viral Transport Medium (UTM) (Copan, Brescia, Italy) were used for nucleic acid extraction and purification with the KingFisher™ Flex system (Thermo Fisher Scientific, Monza, Italy) using the MVP_2Wash_200_Flex program, according to the manufacturer’s instructions. The extracted RNA was eluted in a final volume of 50 µL and stored at –80 °C until use.

SARS-CoV-2 RT-qPCR was performed in BSL-2 labs using the TaqPath COVID-19 CE-IVD RT-PCR Kit (Thermo Fisher Scientific, Waltham, MA, USA), which simultaneously amplifies three viral targets, the ORF1ab gene (FAM), N protein (VIC), and S protein (ABY); MS2 phage (JUN) is detected as the internal positive control. The amplification was carried out in a final volume of 25 μL, which included 5 μL of template, TaqPath 1-Step Multiplex Master Mix (4×), and COVID-19 Real-Time PCR Assay Multiplex, which contains probes and specific primer sets for different SARS-CoV-2 and internal control genomic regions. Each experiment also included TaqPath COVID-19 IVT RNA as a positive control and a negative control. The thermal cycling conditions consisted of an initial Uracil-DNA glycosylase (UNG) incubation step at 25 °C for 2 min, reverse transcription at 53 °C for 10 min, and an initial denaturation and enzyme activation step at 95 °C for 2 min, followed by 40 cycles of denaturation at 95 °C for 3 s, and annealing/extension at 60 °C for 30 s. RT-qPCR was performed on a 7500 Fast Real-Time PCR system (Applied Biosystems, Foster City, CA, USA).

### 2.3. SARS-CoV-2 Immunoassays

All serum samples were first screened for the qualitative detection of anti-SARS-CoV-2 antibodies using the Elecsys^®^ Anti-SARS-CoV-2 test, an electrochemiluminescence immunoassay (Roche Diagnostics, Basel, Switzerland), using a Cobas e 411 instrument. The assay uses a recombinant protein representing the nucleocapsid (N) antigen in a double-antigen sandwich assay format, which favours the detection of high-affinity antibodies against SARS-CoV-2. All serum samples were then quantified using the Roche Elecsys^®^ Anti-SARS-CoV-2 S test for the SARS-CoV-2 spike receptor binding domain. This test enables the determination of both the presence and level of antibodies against SARS-CoV-2 in serum. Both immunoassays were performed according to the manufacturer’s instructions. The efficiency of these kits was determined using standardised controls for each experiment. The results were reported as values of the cut-off index (COI, signal sample/cut-off). All samples were considered negative for anti-SARS-CoV-2 N antibodies, with a COI value < 1.0. As reported above, anti-SARS-CoV-2 N antibodies in the sera were quantified using the Elecsys^®^ Anti-SARS-CoV-2 S test. The total antibody content in the sample was expressed as U/mL, traceable to the Roche Diagnostics internal standard cut-off for anti-SARS-CoV-2 S, which was 0.8 U/mL.

Both tests were developed for human testing, but the double-antigen method is species-independent, as was previously reported [17].

### 2.4. Microneutralisation Test (MTN) for SARS-CoV-2 and BCoV

#### 2.4.1. MTN for SARS-CoV-2

The MTN for SARS-CoV-2 was performed in BSL-3 laboratories. All samples were first heat-inactivated by incubation at 56 °C for 30 min, and then 2-fold dilutions were prepared in Dulbecco’s modified Eagle’s medium (DMEM). The MTN was performed using a modification of a previously described protocol [18]. Briefly, serum samples were initially diluted 1:10, and then 50 μL per well of each serum sample, in duplicate, was subjected to 2-fold serial dilutions (1:20–1:320) in 50 μL of culture medium. Then, 50 μL of a 100 TCID_50_ of hCoV-19/Italy/CAM-INMI-32803-66/2020 (EPI_ISL_493333) was added to each well containing the serially diluted serum and incubated for 1 h at 37 °C. Finally, 100 μL of Vero E6 cell suspension, adjusted with maintenance medium to 10^5^ cells/mL, was added to each well and incubated at 37 °C and 5% CO_2_. After 4–5 days of incubation, the cells were fixed and stained with a solution of 0.1% crystal violet in 5% paraformaldehyde (PFA) for 30 min to detect cytopathic effects (CPE).

The neutralisation endpoint titre was determined as the highest serum dilution at which at least 50% of the wells showed CPE. An MNT titre ≥20 was considered positive [16].

#### 2.4.2. MTN for BCoV

MTN was performed by modifying the protocols previously described [19,20]. Advanced RPMI 1640 (1×; Gibco Ref 12633-012) was used in this protocol.

Samples were heat-inactivated by incubation at 56 °C for 30 min, and then 50 μL per well of serum sample in duplicate was subjected to 2-fold serial dilutions from 1:20 through 1:320, and from 1:4 through 1:64 in 50 μL culture media.

Each dilution was mixed with an equal volume of a viral suspension containing 100 TCID_50_ of BCoV strain Nebraska, which was kindly provided by Dr. S. Reiche (Friedrich-Loeffler-Institut, Insel Reims, Germany) [21], and incubated for 1 h at 37 C. Finally, 100 μL of HRT-18 (a cell line derived from human rectal adenocarcinoma) cell suspension, adjusted with maintenance medium to 10^5^ cells/mL, was added to each well and cultured in 5% CO_2_ at 37 °C_._ After 6–7 days of incubation, the cells were fixed and stained with a 0.1% crystal violet solution in 5% PFA for 30 min to detect cytopathic effects. The neutralisation endpoint titre was determined as the endpoint serum dilution that inhibited BCoV-induced CPE in at least two out of three parallel wells. The complete inhibition of virus propagation in an individual well was accepted as a positive result [20].

### 2.5. Data Analysis

The percentage (with 95% CI) of the results was calculated. Based on age, cows were divided into three age groups: 1–5 years, 6–10 years, and >10 years. Fisher’s exact test was used to compare the age and pregnancy status of the cows with neutralising antibodies. The correlation between antibodies analysed and age was evaluated via Pearson correlation analysis using GraphPad Software InStat 3, (Dotmatics, Atlanta, GA, USA). *p* values less than 0.05 were considered statistically significant.

## 3. Results

To evaluate viral spread, viremia, and seroconversion, nasal and rectal swabs and serum and milk samples were analysed. Milk samples were collected from 21 out of 24 cows due to the presence of two primiparous cows (6 and 10) and a dry cow (19) (Table 1).

All nasal and rectal swabs and milk samples tested negative for SARS-CoV-2 RNA. The results of the analyses performed on the serum samples are reported in Table 1. All tested samples were BCoV-negative (MTN ≥ 4). Eight of the 24 samples (33%, 95% CI 14–52) were negative for SARS-CoV-2 antibody detection. Of the 24 serum samples, 11 (46%, 95% CI 26–66) showed antibodies for SARS-CoV-2 nucleocapsid protein (range: 1.38–7.40), 14 (58%, 95% CI 38–78) showed antibodies for SARS-CoV-2 spike protein (range: 1.60–249.00), and 13 (54%, 95% CI 34–74) developed SARS-COV-2-neutralising antibodies, with titres ranging from 1:20 to 1:160 (Table 1). Interestingly, six of the samples were from pregnant cows (46%, 95% CI 19–73) (5, 12, 17, 19, 20, and 23) (Table 1). Of the 13 animals with neutralising antibodies, 2 of 13 were aged 1–5 years (15%, 95% CI 5–49), 6 of 13 were aged 6–10 years (46%, 95% CI 36–98), and 5 of 13 were aged >10 years (38%, 95% CI 53–113) (Table 2). Of the six pregnant animals with neutralising antibodies, one was aged 1–5 years (17%, 95% CI 9–31), four were aged 6–10 years (67%, 95% CI 12–76), and one was aged over 10 years (17%, 95% CI 13–47) (Table 2).

The correlation between the presence of SARS-CoV-2-neutralising antibodies and the age of the cows was analysed using Fisher’s exact test, which showed a significant relationship (*p* < 0.05). Comparison of the 1–5-year-old group to the >10-year-old group yielded a statistically significant two-sided *p* value (*p* = 0.041). However, no statistically significant difference was observed when comparing the other groups. In addition, no correlation with pregnancy status was detected.

Furthermore, evaluating the analysed antibodies and age of the cows via Pearson’s correlation analysis showed a significant relationship (*p* < 0.05) between anti-S and neutralising antibodies against SARS-CoV-2 with age (Table 3) and, interestingly, Pearson’s correlation analysis showed a strongly significant correlation (*p* < 0.01) between anti-S and anti-N antibodies (Table 3).

None of the cows with neutralising antibodies displayed fever, diarrhoea, and/or respiratory signs at sampling.

## 4. Discussion

The role of cattle in SARS-CoV-2 transmission remains unclear. Although cattle are potentially sensitive to SARS-CoV-2, this study aimed to determine the susceptibility of cows to active SARS-CoV-2 infection at a farm where there was a COVID-19 outbreak among the farmworkers. SARS-CoV-2 and BCoV are both betacoronaviruses, and BCoV is very similar to human coronavirus (HCoV) OC43 (Betacoronavirus 1) [22]. Thus, after the samples tested positive for SARS-CoV-2, they were also analysed for BCoV.

In addition to seroneutralisation, the other methods used for the diagnosis of SARS-CoV-2 include PCR (to amplify and quantify nucleic acids) and new ELISA tests (using serum from infected or vaccinated animals), which allow the detection of antibodies in a wide range of animal species or the discovery of reservoirs or intermediate hosts [23]. In our study, we introduced chemiluminescence, a methodology mainly used for the qualitative and quantitative evaluation of the antibody response in humans to infection with the wild strain of SARS-CoV-2 and to the vaccine antigen, specifically anti-S (quantitative). Using this test, our results showed that 15 of 24 cows exhibited antibodies for SARS-CoV-2, which was the only betacoronavirus detected by serological tests. In some serum samples, the presence of virus neutralisation titres in the absence of detectable antibodies to the spike protein was detected by us (Table 1). According to other authors, in humans, it has been detected that positive antibody amounts against the S-protein of SARS-CoV-2 display weak agreement with virus neutralisation titres [24,25,26]. However, knowledge of the immunological response in cattle is very limited. We believe that our findings are consistent with results of the experimental infection of cattle with SARS-CoV-2 [15]. Of six inoculated animals, the authors detected viral RNA in only two of the inoculated animals, on day 3 after infection. Thus, we presume that only serological positivity was detected because seroconversion had already occurred when we collected swabs.

The limited data on cattle, including our results, indicate that cattle show low susceptibility to SARS-CoV-2 and probably do not function as reservoirs. However, we suppose that in areas with large cattle populations and a high prevalence of SARS-CoV-2 infection in humans, close contact between livestock and farmworkers may cause reverse zoonotic infections in cattle, as has already been described for highly sensitive animal species, such as minks, cats, and dogs [10,13,27].

Interestingly, in our study, Pearson’s correlation analysis showed a strongly significant relationship (*p* < 0.01) between anti-S and anti-N antibodies, and a significant correlation (*p* < 0.05) between anti-S and neutralising antibodies against SARS-CoV-2 with age. In particular, of the 13 cows with neutralising antibodies to SARS-CoV-2, only two were young animals (aged 1–5 years), whereas 11 were adults (aged 6–10 years); this significant difference is similar to reports of SARS-CoV-2 infections in humans. This report describes a COVID-19 pandemic situation that occurred in April 2020, before the detection of SARS-CoV-2 variants of concern by the World Health Organization, such as the alpha-variant (B.1.1.7), which was documented to emerge in autumn 2020 (**Coronavirus Disease** (**COVID-19**) **Situation Reports** (**who.int**)). In this context, in humans, children seemed to be less susceptible to SARS-CoV-2 than adults by wild-type virus than by SARS-CoV-2 variants of concern. This could be a result of several factors, such as the decline in immune protection due to aging, modulation of angiotensin converting enzyme 2 (ACE2) receptor expression, and previous human-CoV infections [28,29,30,31,32].

Of the 13 cows with neutralising antibodies against SARS-CoV-2, six were pregnant. Further studies are required to evaluate this interesting result. To date, there are very little data on COVID-19 during pregnancy and vertical transmission in animals. For example, adult white-tailed deer are highly susceptible to SARS-CoV-2 infection and can transmit the virus vertically [33]. Thus, there are concerns about the risk of neonatal infections in the postpartum phases [34].

The data from the current study indicate that cattle do not seem to be vehicles for the transmission of SARS-CoV-2. Indeed, after the experimental infection of a group of calves with SARS-CoV-2 by intranasal inoculation, no intraspecies transmission of the virus to uninoculated cattle that were in contact with inoculated cattle was detected at 24 h post infection [15]. Therefore, based on the results of our study, we conclude that there is no indication that cattle have played a role in the SARS-CoV-2 human pandemic.

The susceptibility of various animal species to SARS-CoV-2 is of great interest to the international scientific community, and it has been hypothesised that the host range of SARS-CoV-2 may depend on the interaction of the virus spike protein with host cell receptors. ACE2 plays a crucial role in the host cell entry of the virus. Based on phylogenetic and expression pattern analyses of ACE2, various mammals may be susceptible to SARS-CoV-2. However, the amino acid sequence of ACE2 is highly conserved in cattle (83% homology) [35] and cattle share four of the five hotspot residues with humans, suggesting a good probability of interaction between ACE2 and the spike protein of SARS-CoV-2 [36]. Other studies confirmed these data [37,38,39]. In humans, ACE2 is mainly expressed by the epithelial cells of the lung, intestine, kidney, heart, and blood vessels [36]. In contrast, ACE2 receptor expression in cattle is only moderate in the lungs but is higher in the liver and the kidneys [40].

Despite earlier reports on SARS-CoV-2 replication in respiratory ex vivo organ cultures of cattle [41] and the detection of low viral RNA levels after the experimental intranasal inoculation of SARS-CoV-2 in cattle [15], the nasal swabs collected in our study were negative for SARS-CoV-2, probably because seroconversion had already occurred when the nasal swabs were collected by us. Furthermore, the animals tested by us showed no symptoms, and no/mild symptoms have been found during experimental infections [15,16]. A possible explanation may involve the different distributions of ACE2 receptors in cattle. Indeed, a recent investigation established that in cattle (*Bos taurus*), ACE2 was detected in the bronchiolar epithelium in the lungs, but not in the nasal mucosa epithelium [35]. According to Lean et al. (2021), we hypothesise that this pattern of distribution may explain the difference in the susceptibility of animals to SARS-CoV-2.

Despite the negative results from swabs, the serological analyses in our study proved the infection of cattle with SARS-CoV-2 for the first time, although no active viral replication was detected in colostrum-deprived calves that were experimentally infected [16]. To the best of our knowledge, no cases of natural SARS-CoV-2 infection in cattle have been previously reported. As stated above, the serum samples were negative for BCoV infection. Thus, we ruled out the possibility of cross-reactivity due to other betacoronaviruses.

Finally, to avoid economic losses and threats to animal health, biosecurity measures to control SARS-CoV-2 infections may be useful. Conventional measures, including spray disinfection of each vehicle entering the farm, ultraviolet light, and incoming and outgoing showers for service personnel, as well as emerging technological measures, such as electrostatic air filtration systems and heat treatments at high temperatures for disinfection [42], which have been tested on chicken and pig farms in the USA, may be effective methods.

In conclusion, further studies are needed to validate the chemiluminescence technique used to detect SARS-CoV-2 antibodies in cattle, which seemed to seroconvert in the presence of the circulating virus in the farm. The advantages of the chemiluminescence techniques described above are their speed, performance, and the fact that they are inexpensive and can be carried out in BSL-2 labs.

To our knowledge, this is the first report of natural SARS-CoV-2 seroconversion in lactating cows. We hypothesise that this may represent a case of reverse zoonosis. However, the role of cattle in SARS-CoV-2 infection seems to be negligible. Further studies are needed to better define the role of SARS-CoV-2 in cattle, as well as its potential role in the emergence of novel recombinant coronaviruses.

## Figures and Tables

**Table 1:** Results from serum analyses of sampled cows.

Sample ID	Age (In Years)	Ab Anti-N SARS-CoV-2 (r-n COI *)	Ab Anti-S SARS-CoV-2 (U/mL)	MTN ** for SARS-CoV-2	MTN for BCoV
**1**	**3**	**-**	**-**	**-**	**-**
**2**	**8**	**1.45**	**1.74**	**1:80**	**-**
**3**	**6**	**3.57**	**1.41**	**1:40**	**-**
**4**	**5**	**-**	**-**	**-**	**-**
**5**	**10**	**5.02**	**249.00**	**1:160**	-
**6**	**2**	**-**	**-**	**-**	-
**7**	**2**	**-**	**-**	**-**	-
**8**	**11**	**-**	**26.25**	**1:40**	-
**9**	**3**	**5.60**	**219.20**	**1:160**	-
**10**	**2**	**-**	**-**	**-**	-
**11**	**10**	**-**	**3.47**	**-**	-
**12**	**3**	**4.71**	**-**	**1:20**	-
**13**	**2**	**-**	**-**	**-**	-
**14**	**7**	**2.27**	**1.60**	**-**	-
**15**	**11**	**-**	**68.59**	**1:40**	-
**16**	**3**	**-**	**-**	**-**	-
**17**	**7**	**1.38**	**-**	**1:80**	-
**18**	**10**	**-**	**-**	**-**	-
**19**	**10**	**2.21**	**58.66**	**1:40**	-
**20**	**13**	**1.56**	**39.28**	**1:20**	-
**21**	**18**	**2.45**	**176.40**	**1:20**	-
**22**	**9**	**7.40**	**247.50**	**1:80**	-
**23**	**11**	**-**	**80.06**	**1:80**	**-**
**24**	**13**	**-**	**12.04**	**-**	**-**
		**Pos Ab ≥ 1 COI**	**Pos Ab ≥ 0.8 COI**	**Pos MNT ≥ 20**	**Pos MNT ≥ 4**

* COI = cut-off index; ** MNT = microneutralisation test.

**Table 2:** SARS-CoV-2 positivity (MTN) among cows divided into groups by possible risk factor (age and pregnancy status).

Age (Years)	n. Heads	Positivity for SARS-CoV-2	Positive Pregnant Cows
**1–5**	**9**	**2/9** (**22%, 95% CI −5–49**)	**1/6** (**17%, 95% CI −13–47**)
**6–10**	**9**	**6/9** (**67%, 95% CI 36–98**)	**3/6** (**50%, 95% CI 10–90**)
**>10**	**6**	**5/6** (**83%, 95% CI 53–113**)	**2/6** (**33%, 95% CI −5–71**)

**Table 3:** Pearson’s correlation matrix of the age and antibodies (Ab) investigated in cattle.

	Age in Years	Anti-N Ab	Anti-S Ab	Neutralising Ab
**Age (in years)**	1			
**Anti-N Ab**	0.055	1		
**Anti-S Ab**	0.502 *	0.603 **	1	
**Neutralising Ab**	0.487 *	0.334	0.258	1

The significant correlations are indicated in bold. * *p* < 0.05; ** *p* < 0.01.

## Data Availability

The data presented in this study are available on request from the corresponding author.
